# Protein Repeats from First Principles

**DOI:** 10.1038/srep23959

**Published:** 2016-04-05

**Authors:** Pablo Turjanski, R. Gonzalo Parra, Rocío Espada, Verónica Becher, Diego U. Ferreiro

**Affiliations:** 1 Departamento de Computación, Facultad de Ciencias Exactas y Naturales, Universidad de Buenos Aires, Buenos Aires, Argentina; 2 Protein Physiology Lab, Departamento de Química Biológica, Facultad de Ciencias Exactas y Naturales, Universidad de Buenos Aires-CONICET-IQUIBICEN, Buenos Aires, Argentina

## Abstract

Some natural proteins display recurrent structural patterns. Despite being highly similar at the tertiary structure level, repeating patterns within a single repeat protein can be extremely variable at the sequence level. We use a mathematical definition of a repetition and investigate the occurrences of these in sequences of different protein families. We found that long stretches of perfect repetitions are infrequent in individual natural proteins, even for those which are known to fold into structures of recurrent structural motifs. We found that natural repeat proteins are indeed repetitive in their families, exhibiting abundant stretches of 6 amino acids or longer that are perfect repetitions in the reference family. We provide a systematic quantification for this repetitiveness. We show that this form of repetitiveness is not exclusive of repeat proteins, but also occurs in globular domains. A by-product of this work is a fast quantification of the likelihood of a protein to belong to a family.

## Introduction

Several natural proteins are coded with tandem copies of similar amino acid stretches. These molecules are broadly classified according to the length of the minimal repeating unit^1^. Short repetitions of up to five residues usually form fibrillar structures, while repetitions longer than about 60 residues frequently fold as independent globular domains. There is a class of repetitive proteins, repeat proteins, that lays in between these for which folding of the repeating units is coupled and domains are not obvious to define^2^,^3^. Despite being highly similar at the tertiary structure level, repeats within a single protein or in different members of a protein family can be extremely variable at the sequence level^4^, complicating the detection and classification of repeats^1^.

There are many methods to identify repeats in sequences. Some are based on the self-alignment of the primary structure^5^ and others implement spectral analysis of pseudo-chemical characteristics of the amino acids^6^. Since the same structural motif can be encoded by sequences that seem completely unrelated, it is not surprising that alignment-based methods fail to infer true structural repeats. The solutions to find inexact repeats in sequences^7^,^8^ include alphabet replacements using scoring matrices, sophisticated notions of sequence similarity based on an allowed percentage of mismatches, and elaborated mathematical representations such as Hidden Markov Models. To a very large extent these solutions have been satisfactory. However, these methods rely on the fine-tuning of different parameters in order to account for the inexactness of repeats (thresholds for alphabet scoring matrices, allowed percentage of mismatches, e-values for Hidden Markov Models and others). The definition of what constitutes or not a hit for the model remains subject to determination of some threshold.

In this work we turn to “first principles”, using a mathematical definition of repetition, in contrast to the biological repeat, and we consider a repetition finding method with no adjustable parameters. We are not just interested in repetitions, but in *maximal repetitions*, that we will abbreviate as MR. We give the mathematical definition below, but in the context of a protein sequence, a MR is a block of amino acids that occurs two or more times as perfect copies, and any of its extensions (to the left, to the right or both) occurs fewer times. In case that a protein sequence contains a long block that appears twice, as exactly equal copies except for one letter, then we say that there are two repetitions, one to the left and one to the right of that single letter. It is well known that long stretches of perfect repetitions are infrequent in natural proteins, even in those that fold into structures of recurrent structural motifs. However, we observe that a large portion of a protein sequence can be described by stretches of amino acids that occur in other members of a protein family. Thus, a protein family operates as a catalogue of all the possible variations that a block can adopt in any of its members. We quantify how well a given sequence is covered by the repetitions occurring in its family. The method is implemented efficiently by an algorithm with a O(*n* log *n*) computational complexity, where *n* is the size of the protein sequence being tested plus the size of the family dataset. From this quantification we directly obtain a way to decide if one family is more repetitive than another. In addition, this quantification allows us to derive a measure of likelihood for a given sequence to belong to a given family.

### Notation and preliminary definitions

Let 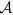 be an alphabet, which is a finite set of symbols. We consider sequences of symbols in 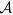. The length of a sequence *s* is denoted by |*s*|. We address the positions of a sequence *s* by counting from 1 to |*s*|. With *s*[*i*..*j*] we denote the sequence that starts in position *i* and ends in position *j* in *s*. If *i* or *j* are out of range then *s*[*i*..*j*] is equal to the empty sequence. We say *u* occurs in *s* if *u* = *s*[*i*..*j*] for some *i*, *j*. In case *s* starts with *u* we say that *s* is an extension of *u*.

**Definition 1 (Gusfield**^8^) A maximal repetition *(MR) is a sequence that occurs more than once in s, and each of its extensions occurs fewer times. We write* 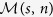 *to denote the set of MRs of lengths greater than or equal to n, that occur in the sequence s.*

**Figure 1:**
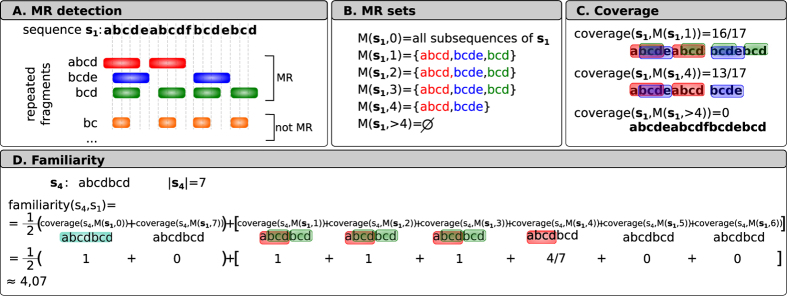
Scheme of the procedure to obtain familiarity function value. (**A**) Maximal repetitions (MR) are computed for input sequence. (**B**) MR sets are filtered by the minimum MR length. (**C**) MR sets are overlapped to input sequence and coverage is calculated. (**D**) *familiarity* is computed based on coverage at every length.

The set of MRs of *s*_1_ = *abcdeabcdfbcdebcd* is {*abcd*, *bcde*, *bcd*}. Observe that *abcd* and *bcde* are the longest MRs, occurring twice. But *bcd* is also a MR because it occurs four times in *s*_1_, and every extension of *bcd* occurs fewer times. On the contrary, *bc* is not a MR because both *bc* and *bcd* occur four times, contradicting the condition that the extension must occur fewer times (see Fig. 1A). The set of MRs of *s*_2_ = *aaaa* is {*aaa*, *aa*, *a*} where *aaa* is a MR occurring twice, *aa* occurs three times and *a* four times. The set of MRs of *s*_3_ = *ab* is empty.

From the given examples is easy to see that MR occurrences can be nested and overlapping. The MRs in any given sequence *s* can have lengths between 1 and a maximum of |*s*| − 1 (this maximum is reached only when the sequence is a chain of the same letter, as *aaaa*). The total number of different MR patterns in a sequence of length |*s*| is at most |*s*| (a mathematical argument ensures that there can be no more different MR patterns than the number of positions in the sequence^9^).

**Definition 2** *Let S be a set of n sequences over the alphabet* 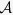
*, S* = {*s*_1_, *s*_2_, … *s*_*n*_}. *The set of MRs in S is the set of MRs of the sequence obtained by concatenation of all sequences in S, interleaved with pairwise different symbols* $_1_, …, $_*n*−1_ *that are not in* 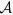. *Thus, the set of MRs in S is the set of MRs in s*_1_$_1_*s*_2_$_2_ … $_*n*−1_*s*_*n*_.

Since each $_*i*_, for *i* = 1, 2, … *n* − 1, occurs only once in the concatenated sequence there will be no MRs containing them. Since the symbols $_*i*_, for *i* = 1, 2, …, *n* − 1, are pairwise different, the set of MRs is invariant respect to the order in which we concatenate the sequences *s*_1_, *s*_2_, …, *s*_*n*_. Concatenation of any permutation of the sequences *s*_1_, …, *s*_*n*_ produces the same set of MRs. Observe that finding the set of MRs in a set of sequences requires more than treating them individually. If *s* and *t* are two sequences and $ is a symbol not occurring in *s* nor in *t*, the MRs in *w* = *s*$*t* may be different from getting the individual MRs and take their union, because a repeat in *w* may occur only once in *s* and only once in *t*.

#### The familiarity function

We define the familiarity function that measures how much of a given protein sequence is covered by MRs from certain family. The greater the familiarity value, the more likely it is for the protein sequence to belong to the family. We first introduce the classical notion of *coverage* of a sequence by a set of MRs, which measures the number of positions in the sequence that are covered by the MRs in the set. We write 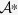 for the set of all sequences over 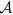, and 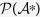 the set of all the parts of 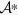, which represents the collection of all the different sets of sequences over 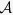. As usual, we write 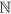 and 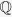 for the set of natural and rational numbers, respectively.

**Definition 3** *The* 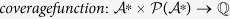 *is such that for any sequence s and any set of sequences R*,


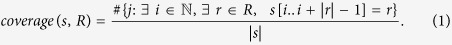


*Thus, coverage*(*s*, *R*) *is a rational number between* 0 *and* 1.

For example, for *s*_1_ = *abcdeabcdfbcdebcd* and 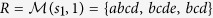 we have *coverage*(*s*_1_, *R*) = 16/17 ≈ 0.94 (see Fig. 1C).

The *familiarity* function measures how much of a sequence is covered by a set of MRs that occur in a family.

**Definition 4** *The familiarity function*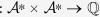 *is defined as follows. For any sequence s and any sequence t*,


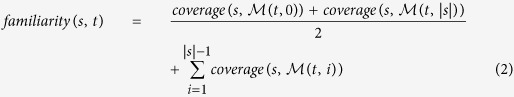


Note that *familiarity* (*s*, *t*) uses 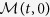 which, by definition, gives all the blocks of the sequence *t*. Thus, for every sequence *s* and *t* the function *familiarity* (*s*, *t*) is a number between 0 and |*s*|. For example, the *familiarity* (*s*_4_, *s*_1_) of *s*_4_ = *abcdbcd* and *s*_1_ = *abcdeabcdfbcdebcd* is around 4.07 because the set of MRs of *s*_1_ is {*abcd*, *bcde*, *bcd*}, and then 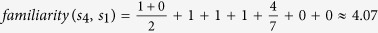 (see Fig. 1D).

If the *familiarity* function is evaluated with the same sequence in the two arguments, *familiarity* (*s*, *s*) the result tells how much of the sequence *s* is covered by its own MRs. For example, the *familiarity*(*s*_5_, *s*_5_) of *s*_5_ = *abcabca* is 4.5 because the set of MRs of *s*_5_ is {*a*, *abca*}, and then 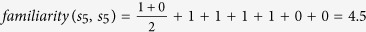. In the case of *s*_6_ = *aaaaaaa* the *familiarity* (*s*_6_, *s*_6_) is 6.5 because the set of MRs of *s*_6_ is {*a*, *aa*, *aaa*, *aaaa*, *aaaaa*, *aaaaaa*}, and then 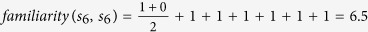. In these examples, *s*_6_ reaches a higher coverage than *s*_5_ when using MRs internal to each of them.

For a given set of sequences, let *t* be the concatenation of its elements separated by pairwise different symbols. Then, *familiarity* (*s*, *t*) indicates how much of the sequence *s* coincides with the MRs in *t*. For example, the *familiarity* (*s*_5_, *t*_1_) of *s*_5_ = *abcabca* and *t*_1_ = *aa*$_1_*ab*$_2_*adddd*$_3_*bca* is around 1.21 because the set of MRs of *t*_1_ is {*a*, *b*, *d*, *dd*, *ddd*}, and then 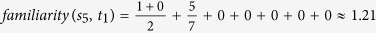. Hereafter we will just use the name of a family in the second argument of the *familiarity* function, to denote the concatenation of all the sequences present in that family, separated by pairwise different symbols.

## Results and Discussion

### Maximal repetitions inside protein sequences

**Figure 2:**
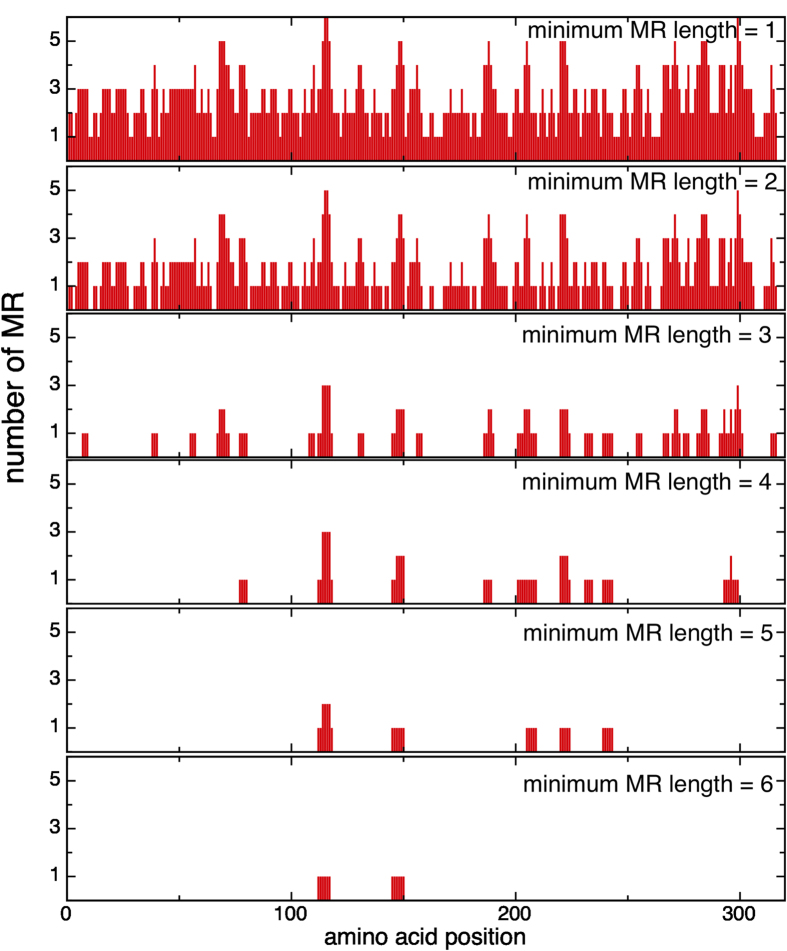
Number of maximal repetitions (MR) that affect each position on a trial sequence. The I*κ*B*α* protein sequence was used as input, the MR set were computed by 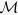
(I*κ*B*α*, *i*) with *i* = 1 … 6. The panels show the counts per position for the different MR sets sorted by minimum length.

Since some proteins contain visible repetitive motifs in structure, we wondered how much of that repetitiveness is maintained at the sequence level. We analyzed the occurrence of exact repetitions on members of the Ankyrin repeat protein family (ANK_*t*_), for which many structures have been solved. Ankyrins constitute the most abundant class of natural repeat proteins, and have been extensively studied^10^. We computed the MRs inside each protein, for all possible lengths (from a minimum length of 1 to the maximum possible, the length of the sequence minus 1). Figure 2 shows the coverage given by MRs inside *s* = *IκBα*(*Uniprot ID: P25963*), a member of the ANK_*t*_, for different minimum MRs lengths (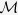
(I*κ*B*α*, *i*) for *i* = 1, …, 6). We found that this protein has 102 MRs distributed as follows: 20 MRs of length 1, 65 MRs of length 2, 11 MRs of length 3, 3 MRs of length 4, 2 MRs of length 5 and only one MR of length 6. The detected MRs are not evenly distributed along the sequence but clustered at specific positions. In most cases the shorter MRs are nested within longer MRs. Moreover, several MRs occur in the same parts of the sequence. These are overlapping occurrences of MRs.

**Figure 3:**
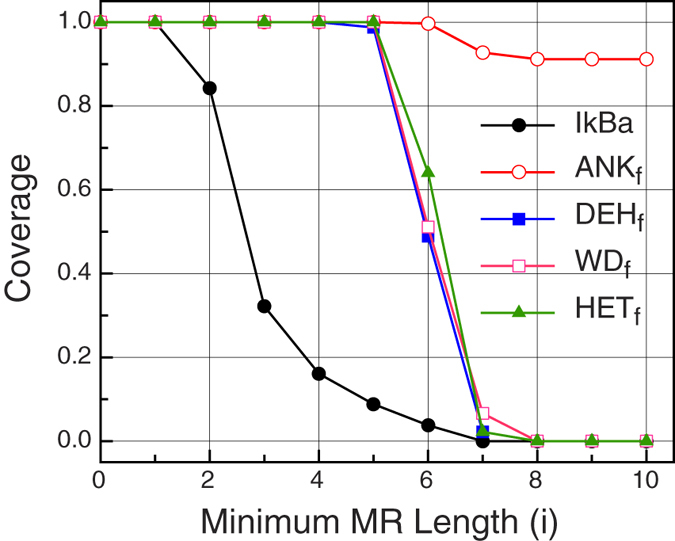
Coverage of a trial sequence of I*κ*B*α* using different MR sets. MR sets were computed from the sequence itself (black) or using groups of sequences derived from distinct families. Values come from applying 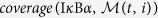 function for *t* = I*κ*B*α*, ANK_*f*_, DEH_*f*_, WD_*f*_, HET_*f*_ and *i* = 0, ..., 10.

We analyzed the coverage of the primary structure of the protein I*κ*B*α* using sets of MRs of increasing minimum length (Fig. 3, black dots). Trivially, the coverage is maximum when MRs of length 1 are considered, because in general every amino acid occurs at least twice inside the protein and then, every position in the protein is covered by some MR of length 1. The coverage is reduced as the minimum MR length is increased, reaching 0 for the values of *i* = 7, …, |*s*|, as there are no exact repetitions larger than or equal to 7 residues. Coverage values for all the members of the ANK_*t*_ were calculated for maximal lengths *i* = 0, …, 10 (see Supplementary Table S1). For each ANK_*t*_ protein, the set *M*(*s*, 1) produces almost full coverage (the coverage function is ≃1). However, the set of MRs of length *i* decays rapidly as *i* increases, and very soon the set of MRs becomes empty. The set of MRs of lengths *i* >= 6 contrasts with the repetitions that can be found in structures, where no sequence information is taken into account^3^. In general, most of the Ankyrin repeat proteins (ANKs) analyzed in this work, are almost entirely covered by structural repeats.

There is a subtype of ANK proteins, which are synthetic constructs composed of (nearly) identical repetitions, for which, as expected, we detected long MRs in sequence. The molecules, such as DARPINs, OR264, OR266 and NRC, have a much larger coverage than natural ANKs, realized by their long perfect repeats (which are directly connected to the construction methods)^11^,^12^ (see Supplementary Table S1).

The results we obtained for ANK_*t*_ were contrasted with results of two other protein families. WD-40 proteins (WD_*t*_) are non-solenoid repetitive proteins with mainly *β* composition, that fold into a globular-like *β*-propeller fold. For these proteins, the distribution of MR lengths is similar to that of ANKs, with infrequent exact repetitions larger than 6 residues (see Supplementary Table S3). Additionally we tested our program in a non-repetitive globular scaffold using members from the Dehalogenase family (DEH_*t*_). Results are shown in Supplementary Table S2. Analogously to repeat proteins, the *coverage* function decreases as the minimum length of the MR considered (*i*) increases. But in this globular family, the *coverage* function reaches zero for lower *i* values (indicating that the MRs in DEH_*t*_ proteins are shorter than the MRs in repeat proteins). The low coverage of the sequences may be consequence of their divergence during evolutionary time scales. Most of existing methods for repeat detection in protein sequences partially fail when proteins are too divergent with respect to a consensus sequence. However, the identification of the individual occurrences of repeats is simple to observe at the structural level. The higher conservation of the repetitive patterns in structures has been recently exploited to visually classify and annotate these kind of proteins^13^. Since protein sequences encode protein structures, we believe there must be a way to unravel the sequence repetitiveness despite the dissimilarity among the repeats.

### Maximal repetitions in protein families

As we have seen previously, long stretches of perfect repetitions are infrequent in natural proteins, even for those which are known to fold into structures of recurrent structural motifs. Sequence-wise, repeats are known to be imperfect. Unfortunately, the methods that assume repeats to be degenerated fail to make a complete detection. Also these methods do not allow to conclude if some individual motifs actually occur or not. For instance, in ANKs, there are some specific sub-motifs that are characteristic of the family when looking at the statistical profile of ANK repeats, as a TPLH motif and variations of it; however, when looking at particular individual sequences it is hard to say whether they correspond to ANK instances or not. All possible variations of typical blocks should be represented in at least one member of the family. Sequence statistical profiles, usually assume that positions are independent. Therefore, when combining different amino acids at adjacent positions, blocks that are not representative of the family can be constructed, since natural covariations are not taken into account. The opposite, i.e. natural occurring blocks that are a consequence of combinations of amino acids with low frequencies may not be detected as part of the motif. We overcome this problem by looking for natural occurring blocks in members of the family. This additionally solves the problem of position independence since these are implicitly used in the short repetitions.

Given a sequence *s* and a family *f* our method consists in finding the repetitions in the family *f* that have some occurrence in the sequence *s*. We first compute the sets 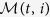, where *t* is the concatenation of the sequences in *f* separated by pairwise different symbols, for all the possible values of *i*, namely *i* goes from 1 to |*s*|. We then compute the coverage made by the elements of 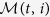 on the sequence *s* using the 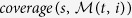 function. As example, Fig. 3 presents the coverage of the I*κ*B*α* protein considering sets of MRs from different families. The coverage was calculated by the *coverage*(I*κ*B*α*, 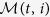) function, using *t* = I*κ*B*α* alone, ANK_*f*_, DEH_*f*_, WD_*f*_ and HET_*f*_ datasets and *i* = 0, …, 10. The HET_*f*_ dataset is a selection of proteins from different families. We observe that, as the minimum MR length increases above *i* = 3 the *coverage*(I*κ*B*α*, 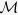
(I*κ*B*α*, *i*)) decays under 0.02 (black line), while the coverage remains close to 1 for MRs detected in larger datasets up to *i* = 6. The coverage only keeps significantly high for longer MRs when using the set of MRs obtained from the ANK family, to which the protein belongs. With these results, we computed the *familiarity* (I*κ*B*α*, *t*) function for *t* = I*κ*B*α* alone, ANK_*f*_, DEH_*f*_, WD_*f*_ and HET_*f*_ datasets. Although the definition of *familiarity* requires the values of 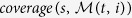 for each *i* in [0..|*s*|], in all the cases we analyzed it was enough to consider *i* just in [0..10], because the coverage for larger values of *i* is negligible. Hereafter we consider the *familiarity* function with lengths *i* ∈ [0..10]. The maximum coverage is obtained for *familiarity* (I*κ*B*α*, ANK_*f*_) = 9.70347. *familiarity* function applied to I*κ*B*α* together with other families have values less than 6.20 (see Supplementary Table S4, Uniprot ID = P25963). This function indicates that I*κ*B*α* belongs to the ANK_*f*_ family.

**Figure 4:**
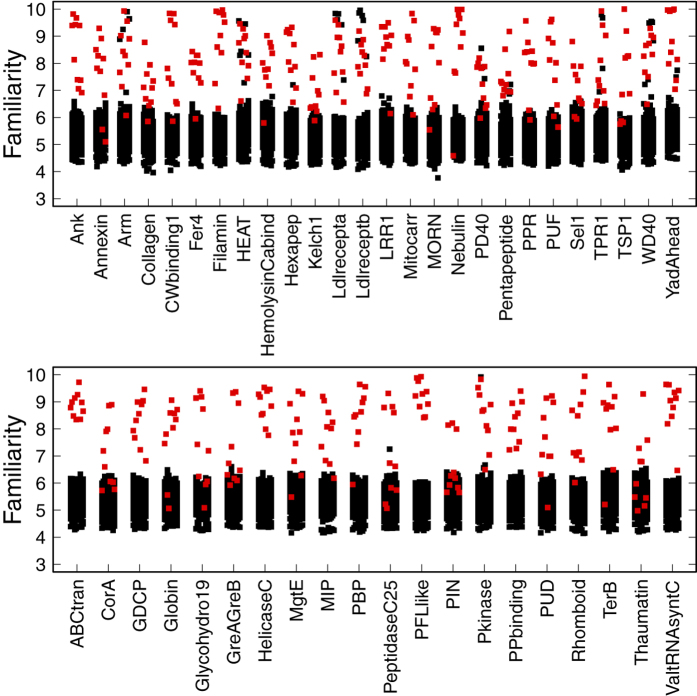
Familiarity of natural protein sequences against the MR of natural protein families. A point denotes a sequence, the x-axis indicates de protein family and the y-axis the familiarity value. When the protein is known to belong to the family indicated n the x-axis, the point is colored in red, otherwise in black. The upper panel contains repeat protein families, and the lower panel contains globular families.

To verify if this hypothesis can be generalized, we extended our analysis through 25 families of repeat proteins and to 20 globular families. For each family we separately detected the set of MRs, excluding ten sequences to be part of a testing set. Afterwards, we calculated the familiarity of every sequence on the testing set against the set of MR of each family. Results can be seen on Fig. 4. A point represents a test sequence. The x-axis indicates the family against which familiarity is evaluated, and the y-axis indicates the value of familiarity of the test sequence to the protein family. When the test sequence belongs to the family indicated in the x-axis, the point is colored red, and black otherwise. In most cases, the highest familiarity values occur when the protein belongs to the testing family. Some exceptions arise when the original family of the protein and the testing family are closely related, such as HEAT and ARM, LDLreceptorA and LDLreceptorB, or WD40 and PD40. Inversely, there are some proteins that obtain a low value of familiarity against the family they belong to (as low as proteins from other families). Many of these testing proteins are multidomain proteins and only a segment of them belongs to the testing family.

To our surprise, we were not able to see differences between the familiarity values of repeat families (upper panel of Fig. 4) and globular families (lower panel of Fig. 4). Our hypothesis is that this high familiarity value between a protein sequence and the set of proteins that constitute the family to which it belongs, is a common feature of protein families that are equilibrated ensembles whose members are mostly composed of exact repetitions ranging from dipeptides to decapeptides. This also suggests that natural proteins are built up from fragments longer than dipeptides but shorter than decapeptides, in line with the general ideas implemented by ‘fragment assembly’ of structural predictions^14^.

There are however, some notable exceptions in the results of familiarity values in our experiments. In the case of ANK_*t*_, protein *P*14585 is composed of more than 1,400 residues, but its ANK region encompasses only about 200 amino acids. As a consequence of this, the coverage (and familiarity) obtained for this sequence in the context of the ANK_*f*_ set does not display values significantly higher than for the other sets corresponding to foreign families.

Other exceptional cases within the ANK_*t*_ group of sequences that are not well explained by MRs found in the ANK_*f*_ set are proteins that fold into ANK-like structures but strongly differ from the rest of the family in their sequence patterns. These cases correspond to sequences *Q*5*ZSV*0 and *Q*5*ZXN*6 from *Legionella sp* and sequence *Q*6*IV*60 which is a viral protein. All these cases are non-eukaryote proteins. The ANK motif is known to be particularly enriched in eukaryotes and within specific eukaryote pathogens (including bacteria and viruses) that use ANK-like proteins to mimic their host counterparts and proceed with the infectious processes^15^. The origin of non-eukaryote ANK-like proteins has been discussed with no consensus about whether they correspond to horizontally transferred molecules with subsequent divergent evolution, or they originated by convergent evolutionary processes.

### How are maximal repetitions distributed in the families?

**Figure 5:**
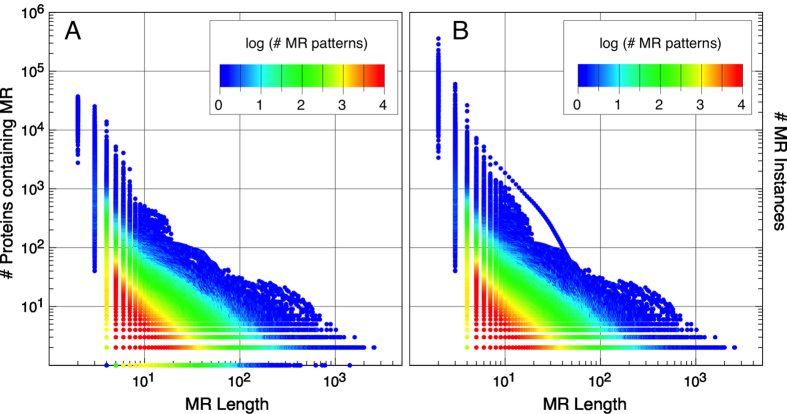
The sequences of the ankyrin family (ANK_*f*_) were used to calculate the maximal repetitions (MR) set. The distribution of the millions of MR found on the whole set is shown according to the length of the pattern. (**A**) Number of different proteins that contain the MR pattern. (**B**) Number of times each MR pattern is present in the whole dataset. The colorscale denotes the number of different MR patterns that occur at a particular coordinate.

For each family dataset (ANK_*f*_, DEH_*f*_, WD_*f*_ and HET_*f*_) we evaluated how its MRs are distributed within the proteins members of the family. We counted how many proteins in the family contain each of the MRs that are found in that family (Fig. 5A shows the case of ANK_*f*_, and Supplementary Fig. S1 shows the case for the remaining datasets). We observe that there is a large number of short MRs occurring in many different proteins (e.g. “TP” appears over 85% of ANK_*f*_ proteins), and a small number of long MRs occurring in just a few different proteins (e.g. “GNPFTPLHCAVINDHE” appears only in two proteins from ANK_*f*_). The longest MR sequence (2,563 residues) has only two instances and appears in two very similar proteins (F1MVI7 and G3MYJ1).

Considering that MRs with lengths *i* >= 6 are found mainly in proteins with respect to their families, we focused on MRs corresponding to those *i* values. ANK_*f*_ has a total of 38,051 proteins but MRs larger than 6 residues do not appear in all of them (the most popular MR of length 6 appears in 4,085 proteins, but in average MRs of this length appear in less than 1,000 proteins). Thus, there is no block larger or equal to six, common to the whole family. The coverage of each member of the ANK family by MRs in ANK_*f*_ comes from many different proteins. In this sense, the ANK proteins seem to be a mosaic of exact MRs that are spread along their whole sequence. The number of mismatches in individual sequences of a given family, found by pairwise alignments inside each sequence, vanishes dramatically if we consider the repeating blocks in other proteins from the family.

In ANK_*f*_ we observe that, starting from MRs of lengths *i* >= 6, Fig. 5A,B are very similar. This shows that MRs occur at most once within each protein sequence. It also proves that long stretches of perfect repetitions are infrequent in natural proteins, even for specific members that are known to fold into structures of recurrent structural motifs. However, there is a collection of recurring MRs that are spread along the members of a family than can be used to (partially) reconstruct any given sequence of the family.

The same analysis over other families (DEH_*f*_ and WD_*f*_) shows similar results to those for ANK_*f*_ (see Supplementary Fig. S1). We also considered a dataset of sequences constructed by scrambling the proteins of a given family (this dataset constructed by permutating the amino acids of each sequence from HET_*f*_ dataset). The result differs completely from the results for actual families (Supplementary Fig. S1). We only found short MRs.

We made the same analysis for the HET_*f*_ dataset (Supplementary Fig. S1). Although the HET_*f*_ has longer MRs than the scrambled protein family dataset, these MRs are notoriously shorter than the MRs in the ANK_*f*_, DEH_*f*_ and WD_*f*_ families. This experiment gives evidence for the difference between sets of sequences that constitute an actual protein family and sets of proteins which do not constitute a family, as compared to sequences that do not correspond to actual proteins.

### Towards a catalogue of repetitions

We computed the set of MRs of length 6 or longer from the ANK_*f*_ dataset, this is 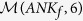. The minimum length value of 6 was selected to be able to compare what was observed in small structural repetitions with a structural tiling methodology^3^. As a result we obtained 4,390,695 MRs with a length of 6 residues which exponentially decreases as the MRs length increases. The most frequent MRs, for instance TPLHLA and GADVNA (and their variants), coincide with the most popular motifs in the ANK HMM profile (Pfam ID: PF00023). We computed the proportion between instances of the MRs and the number of proteins containing them. The most well known motifs, have a proportion close to 1. However, we found several other motifs to be quite popular, as LISHGA, GHLDVV and ELLISH. They have a higher proportion between instances of the MRs and the number of proteins containing them (between 2 and 3), and they are conserved along repeat domains in the ANK_*f*_ dataset. The particularity of these motifs is that their occurrences are not evident when visually observing the sequence logo representation for the ANK HMM profile, because they are composed of highly frequent amino acids at some positions and infrequent amino acids in others. The identified motifs respect the short length covariation in between positions, which is not taken into account in HMMs, in which the positions are assumed to be independent. Consequently, strategies like scanning sequences with HMM profiles need to apply a threshold to accept or not a subsequence as a hit. This can lead to spurious amino acid combinations producing false positives or false negative results. Using short exact sequences in order to look for MRs, considers implicitly the natural covariation among the residues that constitute them and at the same time allows us to avoid the use of thresholds.

## Concluding Remarks

We posed the question: *How repetitive are natural repeat-proteins?* We committed to a mathematical definition of a repetition and found that long stretches of perfect repetitions are infrequent in natural proteins, even for those which are known to fold into structures of recurrent structural motifs. However, we found that repeat proteins have abundant short stretches of amino acids that are perfect repetitions in the reference family. We provided a systematic quantification for this repetitiveness.

Our solution finds all the maximal perfect repetitions, using no adjustable parameters. We use a reference family of protein sequences, that operates as a catalogue of all the possible variations that repeating blocks can adopt. We show that a large portion of each protein sequence can be described by stretches of amino acids occurring in members of the reference family. Thus, each family determines an expected covering of its sequences by family repetitions. This yields a continuous measure of likelihood for any sequence to belong to a given family, quantified by the *familiarity* function. The method could be used as a guiding tool in the design of synthetic proteins, establishing a minimum and a maximum value of a candidate sequence in relation to existing families.

The familiarity function can be implemented with an algorithm whose computational complexity is O(*n* log *n*), where *n* is the size of the protein sequence plus the size of the family dataset. This allows to compute the classification very efficiently.

This work is rooted in the general analysis of 26 repeat protein families and 20 globular protein families, and further focus was applied to the families ANK, WD40 and DEH. The study can be extended to cover the complete protein universe or a substantial part of it. Moreover, the approach does not require a detailed curation of the sequences present in the families. We have limited our current work to the identification of MRs in families and to the computation of the familiarity function. Detailed statistical work remains to be done on MRs in families, such as the average distance between different occurrence of MRs inside the same protein sequence, the number of different MRs per length in each protein sequence. We also suggest to identify the subset of overlapping MRs (and the size of the overlap), the subset of non-overlapping MRs, the subsets of MRs that can be placed one after another, and the subset of MRs that exclude the occurrence of others.

These statistics may yield relations between maximal repeats with some known functional features and to some further conditions for the construction of synthetic proteins.

## Materials and Methods

### Protein family datasets

#### Ankyrin repeat protein (ANK_*f*_) and WD40 repeat protein (WD_*f*_) families datasets

From Uniprot Uniref90 we run hmmsearch from the hmmer suit for a specific HMM family taken from PFAM. Included only sequences that contain at least one hit for the specific family hmm. We excluded those protein sequences containing undefined or ambiguous residues (X, B, Z, J).

#### Haloacid Dehalogenase globular family dataset (DEH_*f*_)

A globular family was retrieved from the SFLD site (http://sfld.rbvi.ucsf.edu/django/superfamily/3/) from which we selected the Haloacid Dehalogenase superfamily.

It was reduced to a 90% identity for non redundancy with cd-hit. Once reduced, the total number of residues in that family was 24,031,515 which was a shorter amount of residues when compared to ANK_*f*_ and WD_*f*_ datasets. In order to be fair with all the datasets and avoid a bias due to random matches product of the dataset size, we reduced the ANK_*f*_ and WD_*f*_ to have an equivalent size to the DEH_*f*_ (~24 M residues).

#### Heterogeneous dataset (HET_*f*_)

We constructed a random dataset by taking a sample of proteins from Uniref90 in such a way that the total size in number of residues was equivalent to the other datasets and the selected proteins do not belong to any of the above mentioned families.

#### Scrambled heterogeneous dataset (HET_*f*_ scrambled)

We constructed a new dataset, scrambling the amino acids of each sequence from HET_*f*_ dataset.

### Protein test groups dataset

#### ANK test group dataset (ANK_*t*_)

We used the set of ANK proteins with known structure, 73 in total, that were analyzed in the publication from Parra and coworkers^16^.

#### WD40 test group dataset (WD_*t*_)

50 Structures corresponding to members of the WD40 Protein Family, not included in the WD_*f*_ set, were randomly selected to conform this group.

#### Haloacid Dehalogenase test group dataset (DEH_*t*_)

50 Structures corresponding to members of the DEH Protein Family were randomly selected to conform this group. These structures were selected from the SFLD site, from those proteins that were not included when building the DEH_*f*_ set.

#### Globular Non Family test group dataset (HET_*t*_)

50 Structures corresponding to a set of unrelated globular proteins was used to conform this group^17^.

### Additional datasets

In order to show that our algorithm is generally applicable we generated datasets for several families both from the repeat and globular types. We retrieved Hidden Markov Models from Pfam for the selected families and searched instances that gave at least one hit to them in the Uniref90 database (a clustered version of UniRef^18^). Since there is not an exhaustive catalogue of repeat proteins we retrieved the Hidden Markov Models for all the repeat protein families that are used in the publication from Espada and coworkers^2^. For the globular proteins we retrieved 20 families from the SCOPe database^19^ selecting representatives for the following classes: a. alpha proteins, b. beta proteins, alpha and beta proteins (c. a/b and d. a+b), and e. membrane and cell surface proteins and peptides. All the selected families are listed in Supplementary Table S5.

### Repeat finding algorithm

The algorithm *findpat*^9^, is the current most efficient algorithm to find exact repetitions (it is particularly well suited for very large inputs). The algorithm requires a parameter *ml* for the minimum length of a MR to be reported, it can be any value greater than or equal to 1. For the special case of *ml* equal to 0 findpat returns all possible blocks of the given sequence. To avoid the use of multiple different special symbols $_*i*_, for as many *i* as needed, we modified the program to have an unique special symbol $ as a symbol that can not be part of MRs. The algorithm *findpat* runs in time *O*(*n* log *n*), where *n* is the length of the whole input (target sequence or sequences for the family of proteins). The code can be downloaded from http://www.dc.uba.ar/people/profesores/becher/software/familiarity.tar.bz2.

## Additional Information

**How to cite this article**: Turjanski, P. *et al.* Protein Repeats from First Principles. *Sci. Rep.* **6**, 23959; doi: 10.1038/srep23959 (2016).

## Supplementary Material

Supplementary Information
