# Supplementary material for: Protein Repeats from First Principles
Source: Sci Rep. 2016 Apr 5;6:23959. doi: 10.1038/srep23959 (PMC4820709; doi:10.1038/srep23959)
Supplement: Supplementary Information [file srep23959-s1.pdf]

Supplementary Information

Protein Repeats from First Principles

Pablo Turjanski[1], R. Gonzalo Parra[2], Rocío Espada[2], Verónica Becher[1] and Diego U. Ferreiro[1,*]

[1]Departamento de Computación, Facultad de Ciencias Exactas y Naturales, Universidad de Buenos Aires, Buenos Aires, Argentina

[2]Protein Physiology Lab, Departamento de Química Biológica, Facultad de Ciencias Exactas y Naturales, Universidad de Buenos Aires-CONICET-IQUIBICEN, Buenos Aires, Argentina

[*]corresponding author: ferreiro@qb.fcen.uba.ar

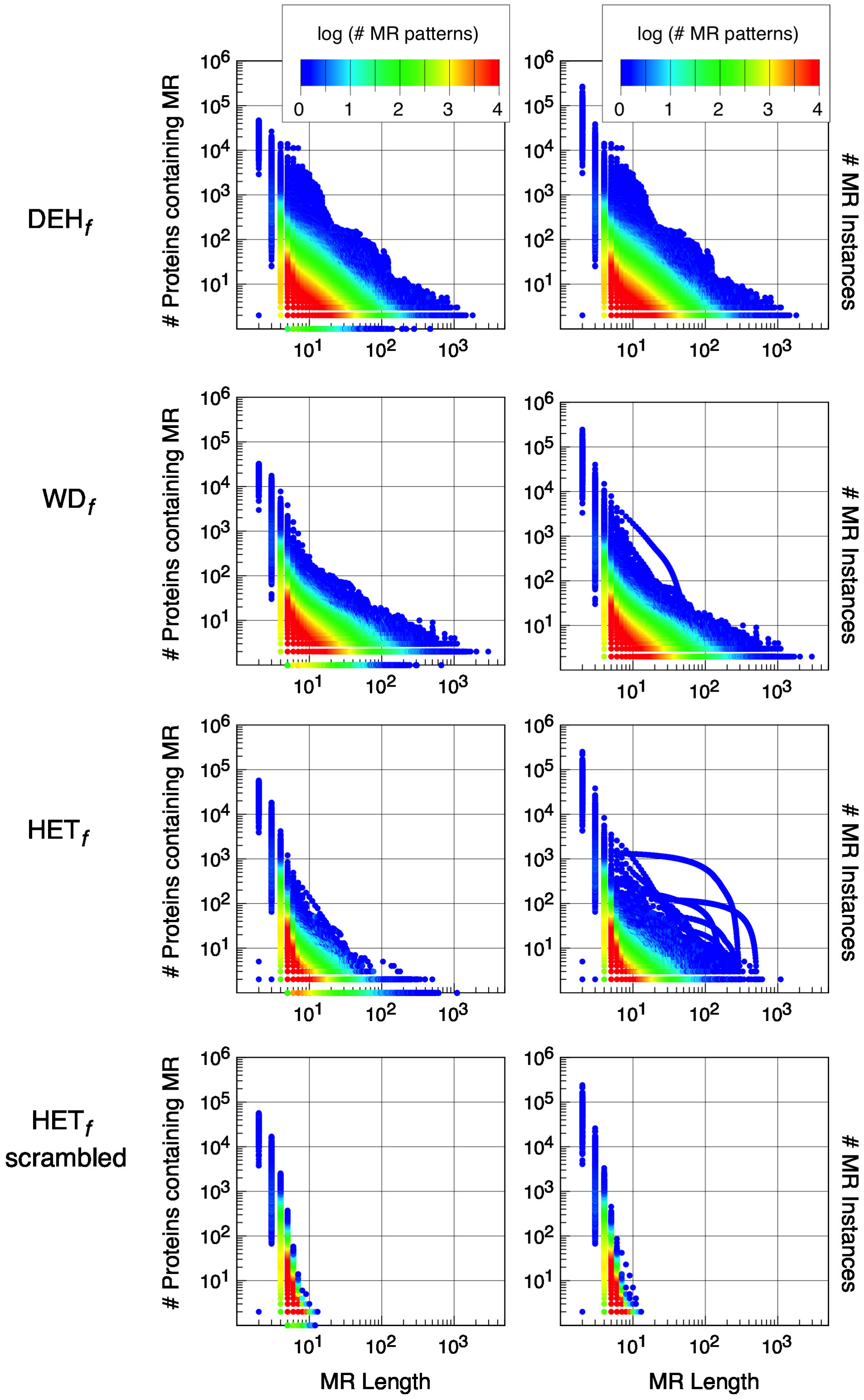

Figure S1: Maximal repeats (MR) distribution within the proteins members of the family. The sequences of distinct families were used to calculate the MR set. The distribution of the millions of MR found on the each set is shown according to the length of the pattern. On the left column, the number of different proteins that contain the MR pattern. On the right column, the number of time each MR pattern is present in the whole dataset. The colorscale denotes the number of different MR patterns that occur at a particular coordinate.

Table S1: Coverage of trial sequences of the ANK$_t$ group. The values for the $coverage(s, \mathcal{M}(s,i))$ function for each sequence, s, name by the UniProt identifier was calculated for different minimum length of the maximal repeats (MR) from $i = 0 \ldots 10$. Values rounded to two decimal places.

**Uniprot ID** (s)	$i=0$	$i=1$	$i=2$	$i=3$	$i=4$	$i=5$	$i=6$	$i=7$	$i=8$	$i=9$	$i=10$
DARPIN-1D5	1.00	0.98	0.79	0.43	0.43	0.43	0.43	0.38	0.27	0.27	0.27
DARPIN-20	1.00	0.99	0.77	0.40	0.40	0.40	0.32	0.27	0.16	0.16	0.16
DARPIN-3ANK	1.00	0.99	0.99	0.99	0.99	0.99	0.99	0.99	0.99	0.99	0.99
DARPIN-3CA1A2N	1.00	0.98	0.72	0.27	0.18	0.00	0.00	0.00	0.00	0.00	0.00
DARPIN-3CA1A2N-OH	1.00	0.98	0.75	0.32	0.24	0.10	0.00	0.00	0.00	0.00	0.00
DARPIN-3H10	1.00	0.99	0.81	0.68	0.60	0.57	0.57	0.52	0.36	0.36	0.36
DARPIN-4ANK	1.00	0.99	0.99	0.99	0.99	0.99	0.99	0.99	0.99	0.99	0.99
DARPIN-AR-3A	1.00	1.00	0.79	0.50	0.50	0.50	0.40	0.32	0.23	0.23	0.23
DARPIN-AR-F8	1.00	0.99	0.80	0.56	0.51	0.51	0.51	0.43	0.30	0.30	0.30
DARPIN-E3-19	1.00	0.99	0.83	0.58	0.55	0.55	0.55	0.55	0.42	0.42	0.30
DARPIN-E3-5	1.00	0.99	0.86	0.60	0.57	0.52	0.52	0.52	0.30	0.30	0.30
DARPIN-H10-2-G3	1.00	0.99	0.81	0.37	0.32	0.32	0.16	0.11	0.00	0.00	0.00
DARPIN-NI1C-Mut4	1.00	0.96	0.73	0.21	0.09	0.00	0.00	0.00	0.00	0.00	0.00
DARPIN-NI3C	1.00	0.99	0.89	0.70	0.68	0.68	0.68	0.68	0.68	0.68	0.68
DARPIN-NI3C-Mut5	1.00	0.99	0.89	0.71	0.67	0.67	0.67	0.67	0.67	0.67	0.67
DARPIN-OFF7	1.00	1.00	0.80	0.51	0.51	0.51	0.51	0.50	0.36	0.26	0.26
E9ADW8	1.00	1.00	0.89	0.26	0.06	0.00	0.00	0.00	0.00	0.00	0.00
NRC	1.00	0.99	0.96	0.90	0.77	0.74	0.69	0.59	0.59	0.45	0.36
O14593	1.00	1.00	0.83	0.26	0.00	0.00	0.00	0.00	0.00	0.00	0.00
O22265	1.00	1.00	0.86	0.26	0.10	0.05	0.02	0.00	0.00	0.00	0.00
O35433	1.00	1.00	0.97	0.37	0.04	0.00	0.00	0.00	0.00	0.00	0.00
O75832	1.00	1.00	0.81	0.16	0.04	0.00	0.00	0.00	0.00	0.00	0.00
OR264	1.00	0.98	0.90	0.81	0.77	0.77	0.70	0.63	0.63	0.63	0.57
OR266	1.00	0.98	0.90	0.78	0.71	0.71	0.61	0.57	0.57	0.57	0.57
P07207	1.00	1.00	1.00	0.81	0.37	0.19	0.14	0.08	0.03	0.01	0.01
P09959	1.00	1.00	0.97	0.50	0.09	0.01	0.00	0.00	0.00	0.00	0.00
P14585	1.00	1.00	1.00	0.58	0.11	0.03	0.01	0.00	0.00	0.00	0.00
P16157	1.00	1.00	0.99	0.75	0.29	0.12	0.08	0.05	0.03	0.00	0.00
P20749	1.00	1.00	0.92	0.49	0.13	0.02	0.00	0.00	0.00	0.00	0.00
P25963	1.00	1.00	0.84	0.32	0.16	0.09	0.04	0.00	0.00	0.00	0.00
P42771	1.00	0.98	0.83	0.22	0.00	0.00	0.00	0.00	0.00	0.00	0.00
P42773	1.00	0.98	0.77	0.07	0.00	0.00	0.00	0.00	0.00	0.00	0.00
P46531	1.00	1.00	1.00	0.83	0.36	0.21	0.09	0.03	0.01	0.01	0.00
P46683	1.00	1.00	0.80	0.07	0.04	0.00	0.00	0.00	0.00	0.00	0.00
P50086	1.00	1.00	0.84	0.20	0.07	0.00	0.00	0.00	0.00	0.00	0.00
P55271	1.00	0.98	0.71	0.16	0.00	0.00	0.00	0.00	0.00	0.00	0.00
P55273	1.00	1.00	0.84	0.23	0.00	0.00	0.00	0.00	0.00	0.00	0.00
P58546	1.00	0.99	0.49	0.15	0.07	0.00	0.00	0.00	0.00	0.00	0.00
P62774	1.00	0.99	0.52	0.15	0.07	0.00	0.00	0.00	0.00	0.00	0.00
P62775	1.00	0.99	0.52	0.15	0.07	0.00	0.00	0.00	0.00	0.00	0.00
Q00420	1.00	1.00	0.89	0.29	0.06	0.00	0.00	0.00	0.00	0.00	0.00

Q01705	1.00	1.00	1.00	0.83	0.37	0.22	0.09	0.04	0.01	0.01	0.00
Q05823	1.00	1.00	0.97	0.51	0.08	0.03	0.00	0.00	0.00	0.00	0.00
Q13418	1.00	1.00	0.92	0.18	0.02	0.00	0.00	0.00	0.00	0.00	0.00
Q13625	1.00	1.00	0.98	0.58	0.12	0.02	0.00	0.00	0.00	0.00	0.00
Q15027	1.00	1.00	0.96	0.44	0.07	0.00	0.00	0.00	0.00	0.00	0.00
Q5ZSV0	1.00	1.00	0.83	0.41	0.25	0.22	0.19	0.17	0.17	0.13	0.13
Q5ZXN6	1.00	1.00	0.99	0.50	0.08	0.01	0.00	0.00	0.00	0.00	0.00
Q60773	1.00	0.99	0.72	0.12	0.05	0.00	0.00	0.00	0.00	0.00	0.00
Q60778	1.00	1.00	0.91	0.36	0.18	0.09	0.05	0.00	0.00	0.00	0.00
Q63ZY3	1.00	1.00	0.97	0.52	0.09	0.03	0.02	0.02	0.00	0.00	0.00
Q6IV60	1.00	1.00	0.84	0.23	0.03	0.00	0.00	0.00	0.00	0.00	0.00
Q6PFX9	1.00	1.00	0.99	0.63	0.33	0.20	0.15	0.14	0.09	0.07	0.06
Q7SIG6	1.00	1.00	0.98	0.50	0.07	0.02	0.00	0.00	0.00	0.00	0.00
Q838Q8	1.00	1.00	0.83	0.32	0.04	0.00	0.00	0.00	0.00	0.00	0.00
Q8IUH5	1.00	1.00	0.94	0.33	0.02	0.00	0.00	0.00	0.00	0.00	0.00
Q8TDY4	1.00	1.00	0.97	0.48	0.04	0.00	0.00	0.00	0.00	0.00	0.00
Q8WUF5	1.00	1.00	0.97	0.56	0.21	0.05	0.03	0.01	0.01	0.01	0.00
Q90623	1.00	1.00	0.99	0.63	0.25	0.09	0.03	0.00	0.00	0.00	0.00
Q91WD2	1.00	1.00	0.97	0.31	0.03	0.02	0.02	0.00	0.00	0.00	0.00
Q92882	1.00	1.00	0.69	0.13	0.00	0.00	0.00	0.00	0.00	0.00	0.00
Q96DX5	1.00	1.00	0.87	0.30	0.05	0.00	0.00	0.00	0.00	0.00	0.00
Q96NW4	1.00	1.00	0.98	0.46	0.11	0.07	0.03	0.02	0.00	0.00	0.00
Q978J0	1.00	0.99	0.85	0.27	0.04	0.00	0.00	0.00	0.00	0.00	0.00
Q99728	1.00	1.00	0.97	0.36	0.06	0.03	0.00	0.00	0.00	0.00	0.00
Q9DFS3	1.00	1.00	0.98	0.44	0.05	0.00	0.00	0.00	0.00	0.00	0.00
Q9H2K2	1.00	1.00	0.99	0.60	0.32	0.24	0.22	0.18	0.16	0.11	0.08
Q9H9B1	1.00	1.00	0.99	0.53	0.10	0.00	0.00	0.00	0.00	0.00	0.00
Q9H9E1	1.00	1.00	0.86	0.19	0.11	0.03	0.00	0.00	0.00	0.00	0.00
Q9HBA0	1.00	1.00	0.98	0.38	0.04	0.01	0.00	0.00	0.00	0.00	0.00
Q9WUD2	1.00	1.00	0.97	0.43	0.07	0.00	0.00	0.00	0.00	0.00	0.00
Q9Y5S1	1.00	1.00	0.96	0.43	0.07	0.00	0.00	0.00	0.00	0.00	0.00
Q9Z2X2	1.00	1.00	0.77	0.19	0.03	0.00	0.00	0.00	0.00	0.00	0.00

Table S2: Coverage of trial sequences of the DEH_t group. The values for the $coverage(s, \mathcal{M}(s,i))$ function for each sequence, s, name by the UniProt identifier was calculated for different minimum length of the maximal repeats (MR) from $i = 0 \ldots 10$. Values rounded to two decimal places.

**Uniprot ID** (s)	$i=0$	$i=1$	$i=2$	$i=3$	$i=4$	$i=5$	$i=6$	$i=7$	$i=8$	$i=9$	$i=10$
A5LNI9	1.00	1.00	0.84	0.23	0.07	0.04	0.00	0.00	0.00	0.00	0.00
B0A4S9	1.00	0.99	0.78	0.17	0.00	0.00	0.00	0.00	0.00	0.00	0.00
B2Z3V8	1.00	1.00	0.69	0.15	0.00	0.00	0.00	0.00	0.00	0.00	0.00
B4A833	1.00	1.00	0.80	0.17	0.00	0.00	0.00	0.00	0.00	0.00	0.00
B4ABS1	1.00	1.00	0.82	0.22	0.03	0.00	0.00	0.00	0.00	0.00	0.00
C2JJ26	1.00	1.00	0.88	0.21	0.09	0.00	0.00	0.00	0.00	0.00	0.00
C6IG92	1.00	0.99	0.56	0.00	0.00	0.00	0.00	0.00	0.00	0.00	0.00
C7RF86	1.00	1.00	0.88	0.27	0.05	0.03	0.00	0.00	0.00	0.00	0.00
D4FVT8	1.00	1.00	0.70	0.00	0.00	0.00	0.00	0.00	0.00	0.00	0.00
D4V4M5	1.00	1.00	0.72	0.20	0.03	0.00	0.00	0.00	0.00	0.00	0.00
D7IJ01	1.00	1.00	0.81	0.17	0.03	0.00	0.00	0.00	0.00	0.00	0.00
D8FP15	1.00	1.00	0.78	0.12	0.00	0.00	0.00	0.00	0.00	0.00	0.00
G9RLJ0	1.00	1.00	0.78	0.09	0.00	0.00	0.00	0.00	0.00	0.00	0.00
K2T267	1.00	0.99	0.73	0.13	0.00	0.00	0.00	0.00	0.00	0.00	0.00
M2DKS0	1.00	0.99	0.89	0.22	0.04	0.00	0.00	0.00	0.00	0.00	0.00
M4SEQ9	1.00	1.00	0.96	0.48	0.05	0.00	0.00	0.00	0.00	0.00	0.00
O08575	1.00	1.00	0.92	0.32	0.07	0.00	0.00	0.00	0.00	0.00	0.00
O15305	1.00	1.00	0.76	0.20	0.03	0.00	0.00	0.00	0.00	0.00	0.00
O29777	1.00	1.00	0.97	0.55	0.10	0.03	0.01	0.00	0.00	0.00	0.00
O32125	1.00	1.00	0.80	0.09	0.02	0.02	0.00	0.00	0.00	0.00	0.00
O32220	1.00	1.00	0.98	0.51	0.10	0.02	0.02	0.02	0.02	0.00	0.00
O59346	1.00	1.00	0.79	0.12	0.00	0.00	0.00	0.00	0.00	0.00	0.00
O67920	1.00	1.00	0.74	0.23	0.00	0.00	0.00	0.00	0.00	0.00	0.00
P05023	1.00	1.00	0.97	0.30	0.08	0.01	0.00	0.00	0.00	0.00	0.00
P06685	1.00	1.00	0.99	0.43	0.05	0.00	0.00	0.00	0.00	0.00	0.00
P0AE22	1.00	1.00	0.73	0.13	0.00	0.00	0.00	0.00	0.00	0.00	0.00
P20649	1.00	1.00	0.99	0.42	0.04	0.01	0.01	0.00	0.00	0.00	0.00
P35670	1.00	0.99	0.72	0.04	0.00	0.00	0.00	0.00	0.00	0.00	0.00
P78330	1.00	1.00	0.75	0.12	0.00	0.00	0.00	0.00	0.00	0.00	0.00
P94592	1.00	1.00	0.83	0.08	0.00	0.00	0.00	0.00	0.00	0.00	0.00
Q04656	1.00	0.97	0.52	0.13	0.07	0.07	0.00	0.00	0.00	0.00	0.00
Q11S56	1.00	1.00	0.77	0.15	0.03	0.00	0.00	0.00	0.00	0.00	0.00
Q2T109	1.00	0.99	0.68	0.02	0.00	0.00	0.00	0.00	0.00	0.00	0.00
Q3UGR5	1.00	1.00	0.83	0.24	0.00	0.00	0.00	0.00	0.00	0.00	0.00
Q5EBQ9	1.00	1.00	0.73	0.06	0.00	0.00	0.00	0.00	0.00	0.00	0.00
Q5SJQ3	1.00	0.99	0.91	0.40	0.18	0.04	0.00	0.00	0.00	0.00	0.00
Q60048	1.00	1.00	0.97	0.45	0.02	0.00	0.00	0.00	0.00	0.00	0.00
Q7ADF8	1.00	1.00	0.75	0.13	0.07	0.00	0.00	0.00	0.00	0.00	0.00
Q7WG29	1.00	1.00	0.63	0.23	0.00	0.00	0.00	0.00	0.00	0.00	0.00
Q8K7R3	1.00	0.99	0.86	0.25	0.03	0.00	0.00	0.00	0.00	0.00	0.00
Q8L1N9	1.00	0.99	0.75	0.25	0.03	0.00	0.00	0.00	0.00	0.00	0.00

Q8TBE9	1.00	1.00	0.81	0.13	0.05	0.02	0.00	0.00	0.00	0.00	0.00
Q96X90	1.00	1.00	0.85	0.21	0.00	0.00	0.00	0.00	0.00	0.00	0.00
Q96XE7	1.00	1.00	0.84	0.13	0.04	0.00	0.00	0.00	0.00	0.00	0.00
Q98I56	1.00	1.00	0.76	0.18	0.04	0.00	0.00	0.00	0.00	0.00	0.00
Q9D020	1.00	1.00	0.85	0.19	0.00	0.00	0.00	0.00	0.00	0.00	0.00
Q9JLV6	1.00	1.00	0.93	0.33	0.02	0.00	0.00	0.00	0.00	0.00	0.00
Q9X0Y1	1.00	1.00	0.86	0.25	0.13	0.05	0.00	0.00	0.00	0.00	0.00
T0QBN5	1.00	1.00	0.79	0.16	0.03	0.00	0.00	0.00	0.00	0.00	0.00
U8H1V1	1.00	1.00	0.73	0.15	0.00	0.00	0.00	0.00	0.00	0.00	0.00

Table S3: Coverage of trial sequences of the WD_t group. The values for the $coverage(s, \mathcal{M}(s,i))$ function for each sequence, s, name by the UniProt identifier was calculated for different minimum length of the maximal repeats (MR) from $i = 0 \ldots 10$. Values rounded to two decimal places.

**Uniprot ID** (s)	$i=0$	$i=1$	$i=2$	$i=3$	$i=4$	$i=5$	$i=6$	$i=7$	$i=8$	$i=9$	$i=10$
A6ZU46	1.00	1.00	0.99	0.44	0.04	0.01	0.00	0.00	0.00	0.00	0.00
O14727	1.00	1.00	0.99	0.52	0.09	0.03	0.00	0.00	0.00	0.00	0.00
O24456	1.00	1.00	0.86	0.39	0.11	0.09	0.04	0.04	0.00	0.00	0.00
O75530	1.00	1.00	0.90	0.10	0.00	0.00	0.00	0.00	0.00	0.00	0.00
O76071	1.00	1.00	0.86	0.37	0.17	0.12	0.08	0.04	0.00	0.00	0.00
O88879	1.00	1.00	0.99	0.53	0.10	0.01	0.00	0.00	0.00	0.00	0.00
O89053	1.00	1.00	0.91	0.28	0.09	0.02	0.00	0.00	0.00	0.00	0.00
P07834	1.00	1.00	0.97	0.50	0.15	0.04	0.02	0.00	0.00	0.00	0.00
P16649	1.00	1.00	0.97	0.53	0.22	0.08	0.05	0.02	0.02	0.00	0.00
P26449	1.00	1.00	0.87	0.17	0.02	0.00	0.00	0.00	0.00	0.00	0.00
P36037	1.00	1.00	0.97	0.39	0.03	0.00	0.00	0.00	0.00	0.00	0.00
P38011	1.00	1.00	0.80	0.40	0.14	0.03	0.00	0.00	0.00	0.00	0.00
P38262	1.00	1.00	0.93	0.36	0.04	0.00	0.00	0.00	0.00	0.00	0.00
P38968	1.00	1.00	0.99	0.61	0.18	0.06	0.03	0.01	0.00	0.00	0.00
P40217	1.00	1.00	0.90	0.22	0.06	0.00	0.00	0.00	0.00	0.00	0.00
P46680	1.00	1.00	0.95	0.36	0.07	0.00	0.00	0.00	0.00	0.00	0.00
P53011	1.00	1.00	0.87	0.18	0.00	0.00	0.00	0.00	0.00	0.00	0.00
P53196	1.00	1.00	0.87	0.17	0.04	0.00	0.00	0.00	0.00	0.00	0.00
P54311	1.00	1.00	0.88	0.28	0.09	0.03	0.00	0.00	0.00	0.00	0.00
P55735	1.00	1.00	0.85	0.26	0.13	0.00	0.00	0.00	0.00	0.00	0.00
P61964	1.00	1.00	0.87	0.43	0.24	0.15	0.10	0.08	0.00	0.00	0.00
P61965	1.00	1.00	0.87	0.43	0.24	0.15	0.10	0.08	0.00	0.00	0.00
P62871	1.00	1.00	0.88	0.28	0.09	0.03	0.00	0.00	0.00	0.00	0.00
P62881	1.00	1.00	0.88	0.30	0.02	0.00	0.00	0.00	0.00	0.00	0.00
P63005	1.00	1.00	0.90	0.34	0.12	0.04	0.04	0.04	0.04	0.04	0.00
P63244	1.00	1.00	0.87	0.31	0.09	0.04	0.04	0.00	0.00	0.00	0.00
P78406	1.00	1.00	0.86	0.15	0.00	0.00	0.00	0.00	0.00	0.00	0.00
P78774	1.00	1.00	0.92	0.23	0.06	0.00	0.00	0.00	0.00	0.00	0.00
P78972	1.00	1.00	0.95	0.32	0.03	0.00	0.00	0.00	0.00	0.00	0.00
Q02793	1.00	1.00	0.87	0.28	0.02	0.00	0.00	0.00	0.00	0.00	0.00
Q03774	1.00	1.00	0.92	0.30	0.01	0.00	0.00	0.00	0.00	0.00	0.00
Q04491	1.00	1.00	0.88	0.28	0.03	0.00	0.00	0.00	0.00	0.00	0.00
Q04724	1.00	1.00	0.97	0.41	0.07	0.01	0.01	0.00	0.00	0.00	0.00
Q05583	1.00	1.00	0.87	0.24	0.05	0.03	0.00	0.00	0.00	0.00	0.00
Q09028	1.00	1.00	0.90	0.30	0.08	0.02	0.00	0.00	0.00	0.00	0.00
Q11176	1.00	1.00	0.97	0.40	0.08	0.00	0.00	0.00	0.00	0.00	0.00
Q13216	1.00	1.00	0.88	0.27	0.04	0.00	0.00	0.00	0.00	0.00	0.00
Q16576	1.00	1.00	0.90	0.24	0.02	0.00	0.00	0.00	0.00	0.00	0.00
Q24572	1.00	1.00	0.90	0.22	0.06	0.02	0.00	0.00	0.00	0.00	0.00
Q24D42	1.00	1.00	0.86	0.12	0.03	0.03	0.00	0.00	0.00	0.00	0.00
Q2YDS1	1.00	1.00	0.93	0.33	0.02	0.02	0.00	0.00	0.00	0.00	0.00

Q58CQ2	1.00	1.00	0.86	0.19	0.02	0.00	0.00	0.00	0.00	0.00	0.00
Q6CN23	1.00	1.00	0.85	0.19	0.05	0.00	0.00	0.00	0.00	0.00	0.00
Q8LNY6	1.00	1.00	0.87	0.29	0.00	0.00	0.00	0.00	0.00	0.00	0.00
Q921E6	1.00	1.00	0.90	0.10	0.00	0.00	0.00	0.00	0.00	0.00	0.00
Q92466	1.00	1.00	0.90	0.14	0.04	0.00	0.00	0.00	0.00	0.00	0.00
Q969H0	1.00	1.00	0.95	0.49	0.14	0.09	0.00	0.00	0.00	0.00	0.00
Q96MX6	1.00	1.00	0.85	0.19	0.02	0.00	0.00	0.00	0.00	0.00	0.00
Q9GZS3	1.00	1.00	0.90	0.30	0.09	0.04	0.04	0.00	0.00	0.00	0.00
Q9Y297	1.00	1.00	0.94	0.35	0.10	0.02	0.00	0.00	0.00	0.00	0.00

Table S4: Values of *familiarity* function for several proteins and families. Values of $familiarity(s,t)$ function for s proteins from ANK_t, DEH_t, HET_t and WD_t test group dataset and t=s, ANK_f, DEH_f, HET_f and WD_f.

**Uniprot ID** (s)	**Test Group**	**familiarity (s,s)**	**familiarity (s,ANK$_f$)**	**familiarity (s,DEH$_f$)**	**familiarity (s,HET$_f$)**	**familiarity (s,WD$_f$)**
DARPIN-1D5	ANK_t	5.06800	9.32400	6.14000	6.42800	6.48400
DARPIN-20	ANK_t	4.47581	9.30242	6.18548	6.48387	6.28226
DARPIN-3ANK	ANK_t	9.89560	9.98901	6.40110	6.47802	7.03846
DARPIN-3CA1A2N	ANK_t	2.64773	9.28977	6.31818	6.59091	6.64773
DARPIN-3CA1A2N-OH	ANK_t	2.89583	8.96875	6.32292	6.63542	6.75000
DARPIN-3H10	ANK_t	6.14231	9.46154	6.15385	6.50000	6.53846
DARPIN-4ANK	ANK_t	9.92460	9.99603	6.38889	6.49206	7.02381
DARPIN-AR-3A	ANK_t	5.08065	8.99677	5.97097	6.40323	6.37097
DARPIN-AR-F8	ANK_t	5.54747	9.43354	5.99367	6.43671	6.43038
DARPIN-E3-19	ANK_t	6.10968	9.10645	5.96452	6.49355	6.54516
DARPIN-E3-5	ANK_t	5.80503	9.18868	6.26730	6.37421	6.48742
DARPIN-H10-2-G3	ANK_t	3.58871	8.81855	6.30645	6.52419	6.30645
DARPIN-NI1C-Mut4	ANK_t	2.47826	9.26630	6.31522	6.69565	6.30435
DARPIN-NI3C	ANK_t	7.50974	9.70130	6.35714	6.71429	6.44805
DARPIN-NI3C-Mut5	ANK_t	7.43949	9.28344	6.44268	6.69108	6.43631
DARPIN-OFF7	ANK_t	5.58599	9.35350	6.09873	6.32166	6.50000
E9ADW8	ANK_t	2.71111	8.22778	6.06111	6.20833	5.99167
NRC	ANK_t	7.35652	8.84783	6.28261	6.30870	6.35217
O14593	ANK_t	2.58462	9.32115	6.10385	6.39231	6.17692
O22265	ANK_t	2.77614	7.50268	6.13539	6.37936	6.34182
O35433	ANK_t	2.88425	8.90811	5.97494	6.18496	6.04177
O75832	ANK_t	2.50885	10.00000	6.03540	6.26549	6.11504
OR264	ANK_t	7.62130	9.04734	6.37574	6.54734	6.48225
OR266	ANK_t	7.19527	9.05621	6.44083	6.57101	6.67160
P07207	ANK_t	4.13559	8.90400	5.84388	6.18054	5.99815
P09959	ANK_t	3.07659	8.64757	6.06663	6.20112	6.09153
P14585	ANK_t	3.22638	6.70644	5.71344	5.99265	5.80861
P16157	ANK_t	3.81366	9.89234	6.03854	6.21983	6.18155
P20749	ANK_t	3.06388	9.63326	6.17401	6.39207	6.32819
P25963	ANK_t	2.95110	9.70347	5.99842	6.16246	6.07729
P42771	ANK_t	2.52564	9.62179	6.39744	6.58974	6.32051
P42773	ANK_t	2.32143	8.86905	6.05357	6.33929	6.21429
P46531	ANK_t	4.04090	9.96732	5.71996	6.17319	5.91957
P46683	ANK_t	2.40500	8.79250	6.07500	6.28000	6.18000
P50086	ANK_t	2.60965	8.88158	5.96053	6.07456	6.04386
P55271	ANK_t	2.35385	9.35769	6.29231	6.53846	6.28462
P55273	ANK_t	2.57229	9.18072	6.48193	6.54217	6.28916
P58546	ANK_t	2.20339	10.00000	6.16949	6.48305	6.16949
P62774	ANK_t	2.22881	9.93220	6.17797	6.48305	6.20339
P62775	ANK_t	2.22881	9.93220	6.17797	6.48305	6.20339
Q00420	ANK_t	2.73760	9.70627	6.10836	6.30679	6.19452

Q01705	ANK$_t$	4.07171	9.23983	5.70348	6.13177	5.89826
Q05823	ANK$_t$	3.10324	8.65992	6.07490	6.25978	6.07760
Q13418	ANK$_t$	2.62168	9.83739	5.95575	6.06637	5.98230
Q13625	ANK$_t$	3.19947	9.97562	6.00621	6.28989	6.09486
Q15027	ANK$_t$	2.97432	9.95946	6.05676	6.33514	6.17568
Q5ZSV0	ANK$_t$	3.94565	6.36957	6.06793	6.32337	6.00000
Q5ZXN6	ANK$_t$	3.08588	6.35195	6.02898	6.27028	6.04162
Q60773	ANK$_t$	2.37952	9.19578	6.36145	6.40964	6.33735
Q60778	ANK$_t$	3.09331	8.98607	6.24373	6.41365	6.23259
Q63ZY3	ANK$_t$	3.14160	9.40071	6.13690	6.36957	6.26028
Q6IV60	ANK$_t$	2.59507	6.33099	6.06338	6.16901	5.98944
Q6PFX9	ANK$_t$	4.12500	9.73977	6.24697	6.40379	6.25152
Q7SIG6	ANK$_t$	3.06994	9.56002	5.99791	6.19833	6.08768
Q838Q8	ANK$_t$	2.68905	8.29104	6.07711	6.26617	6.10199
Q8IUH5	ANK$_t$	2.78956	9.92880	5.83386	5.98892	5.85759
Q8TDY4	ANK$_t$	2.99612	9.72425	6.08250	6.23976	6.20321
Q8WUF5	ANK$_t$	3.36353	9.62077	6.18478	6.43720	6.28684
Q90623	ANK$_t$	3.49203	9.92281	6.23207	6.47809	6.36952
Q91WD2	ANK$_t$	2.83700	9.29780	5.99381	6.21527	5.99794
Q92882	ANK$_t$	2.32243	9.98832	6.14953	6.22897	6.00000
Q96DX5	ANK$_t$	2.72109	9.28231	5.88435	6.14286	6.08844
Q96NW4	ANK$_t$	3.17714	9.90714	5.95238	6.15619	6.07524
Q978J0	ANK$_t$	2.64815	7.31481	6.16138	6.26720	6.08201
Q99728	ANK$_t$	2.90669	9.97748	6.01609	6.12806	6.09974
Q9DFS3	ANK$_t$	2.96596	9.47946	6.05282	6.19131	6.04695
Q9H2K2	ANK$_t$	4.34991	9.93568	6.13036	6.25300	6.13979
Q9H9B1	ANK$_t$	3.11864	9.67989	6.01618	6.16949	6.10015
Q9H9E1	ANK$_t$	2.69169	9.53514	5.89936	6.18690	6.15815
Q9HBA0	ANK$_t$	2.91102	9.75488	6.01665	6.15557	5.99254
Q9WUD2	ANK$_t$	2.97175	8.33903	5.99409	6.18331	6.01511
Q9Y5S1	ANK$_t$	2.95812	9.48429	6.00785	6.21335	6.00524
Q9Z2X2	ANK$_t$	2.49567	9.54545	6.08442	6.24026	6.14069
A5LNI9	DEH$_t$	2.67899	6.07198	9.86965	6.28988	6.11089
B0A4S9	DEH$_t$	2.43678	6.06322	9.90230	6.39080	5.97126
B2Z3V8	DEH$_t$	2.33750	6.00833	7.10417	6.03333	5.96667
B4A833	DEH$_t$	2.46610	6.00847	8.16949	6.23898	5.99492
B4ABS1	DEH$_t$	2.56400	5.93200	9.93400	6.20400	6.08000
C2JJ26	DEH$_t$	2.68750	6.14062	9.41211	6.31250	6.08984
C6IG92	DEH$_t$	2.05396	6.03957	7.30216	6.17626	6.03237
C7RF86	DEH$_t$	2.73298	5.88482	6.77880	6.31152	5.89005
D4FVT8	DEH$_t$	2.19787	5.91702	8.12340	6.09149	6.01489
D4V4M5	DEH$_t$	2.45082	5.96311	8.51639	6.30738	5.99180
D7IJ01	DEH$_t$	2.50388	5.96512	8.57171	6.43023	6.02713
D8FP15	DEH$_t$	2.39121	6.12343	7.89958	6.35774	6.15690
G9RLJ0	DEH$_t$	2.37218	5.92105	9.28947	6.16917	5.92857
K2T267	DEH$_t$	2.35475	6.03631	9.81006	6.46089	6.18715

M2DKS0	DEH_t	2.64786	6.04864	9.79183	6.32101	6.13035
M4SEQ9	DEH_t	2.99457	6.08288	9.59986	6.66372	6.18478
O08575	DEH_t	2.80263	6.07143	10.00000	6.16917	6.06203
O15305	DEH_t	2.49187	5.91463	9.67683	6.17886	5.92276
O29777	DEH_t	3.16667	6.24627	8.13744	6.64677	6.26368
O32125	DEH_t	2.42857	5.92481	9.30639	6.14662	6.10150
O32220	DEH_t	3.18080	6.12095	9.91459	6.71945	6.19202
O59346	DEH_t	2.41286	6.06017	8.80498	6.28423	5.93983
O67920	DEH_t	2.47546	6.24847	7.08896	6.57975	6.23006
P05023	DEH_t	2.87004	6.06241	9.73642	6.50294	6.07269
P06685	DEH_t	2.97801	6.01222	9.84702	6.49316	6.02590
P0AE22	DEH_t	2.35232	6.01477	10.00000	6.19620	6.13291
P20649	DEH_t	2.97102	6.03635	9.51791	6.55901	6.04162
P35670	DEH_t	2.25177	6.08865	9.69858	6.42199	6.34397
P78330	DEH_t	2.35778	6.05111	10.00000	6.26444	6.11333
P94592	DEH_t	2.41579	6.04737	9.47719	6.26491	6.09298
Q04656	DEH_t	2.25556	6.38889	8.45000	6.77778	6.34444
Q11S56	DEH_t	2.43993	5.95230	7.00000	6.20318	6.05124
Q2T109	DEH_t	2.18984	6.21658	9.75134	6.51070	6.16845
Q3UGR5	DEH_t	2.57469	6.20954	9.46888	6.42531	6.11411
Q5EBQ9	DEH_t	2.28302	5.97642	9.93868	6.08019	5.94811
Q5SJQ3	DEH_t	3.01737	6.43436	7.05019	6.71622	6.39961
Q60048	DEH_t	2.94304	6.07243	8.48101	6.42827	6.10197
Q7ADF8	DEH_t	2.45045	6.12613	9.80856	6.46396	6.20721
Q7WG29	DEH_t	2.36034	6.05307	8.79609	6.31006	6.11453
Q8K7R3	DEH_t	2.62992	6.00787	9.36024	6.29921	6.06693
Q8L1N9	DEH_t	2.51961	6.01373	7.91765	6.31569	6.14706
Q8TBE9	DEH_t	2.51481	6.11111	9.65370	6.25185	6.12593
Q96X90	DEH_t	2.56364	6.05909	6.52727	6.37727	6.10455
Q96XE7	DEH_t	2.50971	6.09223	6.55340	6.08738	5.93204
Q98I56	DEH_t	2.46774	6.07604	8.17281	6.43548	6.14055
Q9D020	DEH_t	2.54230	5.97734	9.47281	7.97432	6.00151
Q9JLV6	DEH_t	2.77969	6.06897	9.44636	6.25287	6.12069
Q9X0Y1	DEH_t	2.78704	6.22222	7.67130	6.50926	6.30093
T0QBN5	DEH_t	2.48062	5.98450	9.30620	6.43411	5.96124
U8H1V1	DEH_t	2.38293	6.01220	9.70976	6.24634	6.13415
B1MJ53	HET_t	2.57143	6.10065	6.17208	6.47403	6.10714
D3H0F7	HET_t	2.73485	6.08333	6.15909	6.31061	5.97980
E6Z0R3	HET_t	2.85146	6.10460	6.02092	6.21757	6.03975
F4AR88	HET_t	2.58306	6.07475	6.04485	6.99834	6.10465
O06961	HET_t	2.72886	6.04229	6.11443	6.96891	6.08706
O52806	HET_t	2.34878	5.94390	6.11951	6.38780	6.02195
O58456	HET_t	2.51887	6.06604	6.15283	6.25849	6.05849
P00437	HET_t	2.26569	5.75523	5.78033	6.11925	5.92259
P00693	HET_t	2.74658	5.96575	6.02740	6.31050	6.05251
P00772	HET_t	2.46617	5.90977	5.97368	6.18797	5.92857

P00800	HET_t	2.79197	6.03102	5.95803	6.21350	6.08942
P00918	HET_t	2.41154	5.90385	5.90000	6.09231	5.95385
P02883	HET_t	2.25362	5.79952	5.80435	5.99758	5.82367
P09211	HET_t	2.49524	6.04762	5.99048	6.29524	5.99524
P0A6C8	HET_t	2.61628	6.13953	6.34884	6.70543	6.24031
P0A8M3	HET_t	2.70093	5.91433	5.90343	6.38941	5.81464
P0C0Y9	HET_t	2.53571	5.92857	5.94481	7.42695	6.08442
P0C512	HET_t	2.67610	5.90252	6.02201	6.89203	6.02411
P23472	HET_t	2.58360	6.08842	6.00804	6.21383	5.98553
P23904	HET_t	2.32700	5.79958	5.82489	6.00633	5.91772
P26663	HET_t	3.44219	6.05947	6.01561	6.59319	6.04485
P27448	HET_t	3.01394	6.10823	6.00598	6.63347	6.14542
P29476	HET_t	3.05353	5.96326	5.90308	6.22848	5.95136
P32169	HET_t	2.43431	5.99270	5.85036	6.22993	5.95255
P35202	HET_t	2.46865	6.00157	5.98903	6.16771	5.91066
P37352	HET_t	2.85831	5.94965	6.16745	6.28454	6.04801
P46154	HET_t	2.68296	6.08647	6.08897	6.63283	6.08647
P50586	HET_t	2.77129	6.21485	6.08614	6.33168	6.20693
P61086	HET_t	2.24500	6.20000	6.16500	6.64000	6.08500
P69834	HET_t	2.49338	6.05960	6.12914	6.32781	6.16225
Q26997	HET_t	2.51304	5.89130	5.88696	6.22174	5.94783
Q3IWB0	HET_t	3.08891	6.22658	6.27820	6.52868	6.30497
Q51723	HET_t	2.68644	5.95127	5.97669	6.25424	5.85169
Q54727	HET_t	2.93615	6.02941	6.01793	6.13989	6.02941
Q5TA50	HET_t	2.29907	6.09346	6.14486	6.24299	6.14953
Q5TLG6	HET_t	2.25446	5.73661	5.77232	5.96875	5.80804
Q6DLV0	HET_t	3.47434	5.90531	5.95752	6.16195	5.99115
Q6G441	HET_t	2.62381	6.09762	6.15476	6.74643	6.15952
Q70C53	HET_t	2.64793	5.96746	5.87574	6.03846	5.84911
Q873X9	HET_t	2.76790	6.02194	5.94342	6.26443	6.19515
Q8A7T5	HET_t	2.70487	6.11460	5.97465	6.43306	6.00913
Q8DCF5	HET_t	2.55389	5.96108	6.00000	6.64222	5.97605
Q8TX37	HET_t	2.88268	6.20950	6.29050	6.39385	6.20950
Q97DM1	HET_t	2.62946	5.80394	5.71951	5.98218	5.93715
Q97VM5	HET_t	2.67969	6.13542	6.20573	6.38021	6.20573
Q9IFX1	HET_t	2.94501	5.97826	5.91304	6.19309	6.06522
Q9KFI6	HET_t	2.55152	5.94028	6.01288	6.09719	5.90984
Q9NUI1	HET_t	2.67123	6.16438	6.04795	6.70548	6.30137
Q9P286	HET_t	2.90334	6.15299	5.93115	6.34562	6.12309
Q9WZY5	HET_t	2.49187	5.91463	5.89837	6.28862	5.95122
A6ZU46	WD_t	2.98594	6.11305	5.99606	6.26940	8.46794
O14727	WD_t	3.13301	6.02484	5.91747	6.11538	9.78726
O24456	WD_t	3.03211	6.05046	6.07798	6.16972	8.90214
O75530	WD_t	2.50227	5.93991	6.26417	6.09637	10.00000
O76071	WD_t	3.14012	6.02212	5.83333	6.09587	9.85103
O88879	WD_t	3.12770	6.10689	5.94756	6.16293	8.70536

O89053	WD_t	2.79501	6.11605	5.97722	6.16377	9.34924
P07834	WD_t	3.17522	6.12901	6.09692	6.24968	7.62901
P16649	WD_t	3.38079	6.48177	6.21108	6.46143	8.45863
P26449	WD_t	2.56452	5.97214	5.94575	6.09531	6.52346
P36037	WD_t	2.89441	6.07343	6.02587	6.17552	7.58462
P38011	WD_t	2.86991	6.07680	5.89498	6.20846	9.03762
P38262	WD_t	2.84019	6.05701	6.02523	6.24206	7.56168
P38968	WD_t	3.37510	6.17557	5.97840	6.25805	7.30361
P40217	WD_t	2.67867	5.94092	5.76513	6.11960	9.54755
P46680	WD_t	2.88049	6.03333	5.96016	6.10000	8.23902
P53011	WD_t	2.55444	6.05587	5.85530	6.04728	9.23352
P53196	WD_t	2.57434	5.95564	5.95803	6.21223	6.55276
P54311	WD_t	2.77647	6.04412	5.86765	6.13824	9.99118
P55735	WD_t	2.75155	5.87888	5.80435	5.98137	9.64752
P61964	WD_t	3.37425	6.12874	5.96407	6.09880	10.00000
P61965	WD_t	3.37425	6.12874	5.96407	6.09880	10.00000
P62871	WD_t	2.77647	6.04412	5.86765	6.13824	9.99118
P62881	WD_t	2.69747	5.94051	5.87722	6.14304	9.78101
P63005	WD_t	3.07561	5.94634	5.90732	6.04146	10.00000
P63244	WD_t	2.84700	5.95741	5.85331	6.08044	10.00000
P78406	WD_t	2.50272	5.80978	5.72283	5.97826	10.00000
P78774	WD_t	2.69629	6.03581	5.95093	6.10212	7.21618
P78972	WD_t	2.80533	6.04508	5.89959	6.17828	7.40779
Q02793	WD_t	2.66877	5.99874	5.93073	6.17758	6.61083
Q03774	WD_t	2.73198	5.99550	5.95045	6.06532	7.38063
Q04491	WD_t	2.67845	5.90404	5.91751	6.07912	9.54377
Q04724	WD_t	2.96234	6.08701	5.96883	6.22208	9.81234
Q05583	WD_t	2.69697	6.12121	5.91212	6.21515	7.93485
Q09028	WD_t	2.80118	6.05529	5.99176	6.02706	10.00000
Q11176	WD_t	2.95172	5.99264	5.91899	6.10720	8.57774
Q13216	WD_t	2.69192	5.92424	5.91919	6.04798	9.47727
Q16576	WD_t	2.65059	6.03176	5.99882	6.01294	9.94588
Q24572	WD_t	2.70000	6.00698	5.97674	6.01628	9.47442
Q24D42	WD_t	2.53499	5.83236	5.81195	6.04519	8.56997
Q2YDS1	WD_t	2.79839	6.01815	5.92540	6.19153	7.14516
Q58CQ2	WD_t	2.57527	6.00000	5.92742	6.08333	9.57661
Q6CN23	WD_t	2.57965	5.81858	5.89528	5.98083	6.29941
Q8LNY6	WD_t	2.65526	5.93158	5.90000	6.01053	7.78684
Q921E6	WD_t	2.50227	5.93991	6.26417	6.09637	10.00000
Q92466	WD_t	2.57260	5.97541	5.91920	6.08782	8.87822
Q969H0	WD_t	3.17751	6.16478	6.01061	6.25106	9.48798
Q96MX6	WD_t	2.56162	6.01541	5.81933	6.03782	9.86695
Q9GZS3	WD_t	2.86721	5.95246	6.02131	6.19180	9.97049
Q9Y297	WD_t	2.90992	6.00744	5.93306	6.07190	10.00000

Table S5: Additional datasets. Dataset description for several protein families used in this work.

Family Name	Type	Total number of residues	Pfam ID
ABC_tran	Globular	24,000,412	PF00005
Ank	Repeat	24,000,002	PF00023
Annexin	Repeat	1,327,710	PF00191
Arm	Repeat	14,246,437	PF00514
Collagen	Repeat	6,213,389	PF01391
CorA	Globular	5,855,946	PF01544
CW_binding_1	Repeat	3,070,696	PF01473
Fer4	Repeat	13,342,438	PF00037
Filamin	Repeat	5,278,919	PF00630
GDC-P	Globular	4,904,935	PF02347
Globin	Globular	2,013,817	PF00042
Glyco_hydro_19	Globular	1,099,396	PF00182
GreA_GreB	Globular	1,561,778	PF01272
HEAT	Repeat	24,000,562	PF02985
Helicase_C	Globular	24,001,596	PF00271
HemolysinCabind	Repeat	12,189,037	PF00353
Hexapep	Repeat	10,993,336	PF00132
Kelch_1	Repeat	7,901,965	PF01344
Ldl_recept_a	Repeat	13,481,548	PF00057
Ldl_recept_b	Repeat	5,706,663	PF00058
LRR_1	Repeat	24,000,048	PF00560
MgtE	Globular	2,304,763	PF01769
MIP	Globular	3,232,513	PF00230
Mito_carr	Repeat	9,276,723	PF00153
MORN	Repeat	4,254,637	PF02493
Nebulin	Repeat	1,765,482	PF00880
PBP	Globular	1,475,270	PF01161
PD40	Repeat	22,286,434	PF07676
Pentapeptide	Repeat	5,625,441	PF00805
Peptidase_C25	Globular	1,260,515	PF01364
PFL-like	Globular	1,952,299	PF02901
PIN	Globular	3,151,435	PF01850
Pkinase	Globular	24,000,261	PF00069
PP-binding	Globular	24,000,381	PF00550
PPR	Repeat	24,000,333	PF01535
PUD	Globular	1,173,193	PF03714
PUF	Repeat	2,956,344	PF00806
Rhomboid	Globular	4,786,260	PF01694
Sel1	Repeat	9,677,695	PF08238
TerB	Globular	1,674,918	PF05099
Thaumatin	Globular	828,946	PF00314
TPR_1	Repeat	24,000,070	PF00515
TSP_1	Repeat	11,488,042	PF00090
Val_tRNA-synt_C	Globular	4,098,556	PF10458

WD40	Repeat	24,000,119	PF00400
YadA_head	Repeat	4,451,109	PF05658
